# Association of the prefrailty with global brain atrophy and white matter lesions among cognitively unimpaired older adults: the Nakajima study

**DOI:** 10.1038/s41598-022-16190-7

**Published:** 2022-08-01

**Authors:** Moeko Noguchi-Shinohara, Kenjiro Ono, Sohshi Yuki-Nozaki, Kazuo Iwasa, Masami Yokogawa, Kiyonobu Komai, Benjamin Thyreau, Yasuko Tatewaki, Yasuyuki Taki, Mao Shibata, Tomoyuki Ohara, Jun Hata, Toshiharu Ninomiya, Masahito Yamada

**Affiliations:** 1 Department of Neurology, Kanazawa University Graduate School of Medical Sciences, 13-1 Takara-machi, Kanazawa, 920-8640 Japan; 2 Department of Preemptive Medicine for Dementia, Kanazawa University Graduate School of Medical Sciences, Kanazawa, Japan; 3 Department of Health and Medical Sciences, Ishikawa Prefectural Nursing University, Kahoku, Japan; 4 Division of Health Sciences, Department of Physical Therapy, Kanazawa University Graduate School of Medical Sciences, Kanazawa, Japan; 5 Department of Neurology, Hokuriku Brain and Neuromuscular Disease Center, National Hospital Organization Iou National Hospital, Kanazawa, Japan; 6 Department of Aging Research and Geriatric Medicine, Institute of Development, Aging and Cancer, Tohoku University, Sendai, Japan; 7 Smart-Aging Research Center, Institute of Development, Aging and Cancer, Tohoku University, Sendai, Japan; 8 Department of Geriatric Medicine and Neuroimaging, Tohoku University Hospital, Sendai, Japan; 9 Department of Epidemiology and Public Health, Graduate School of Medical Sciences, Kyushu University, Fukuoka, Japan; 10 Department of Neuropsychiatry, Graduate School of Medical Sciences, Kyusyu University, Fukuoka, Japan; 11 Kudanzaka Hospital, Tokyo, Japan

**Keywords:** Dementia, Magnetic resonance imaging

## Abstract

Physical frailty has been associated with adverse outcomes such as dementia. However, the underlying structural brain abnormalities of physical frailty are unclear. We investigated the relationship between physical frailty and structural brain abnormalities in 670 cognitively unimpaired individuals (mean age 70.1 years). Total brain volume (TBV), hippocampal volume (HV), total white matter hypointensities volume (WMHV), and estimated total intracranial volume (eTIV) on the 3D T1-weighted images were automatically computed using FreeSurfer software. Participants were divided into two states of physical frailty (robust vs. prefrail) based on the revised Japanese version of the Cardiovascular Health Study criteria. The multivariable-adjusted mean values of the TBV-to-eTIV ratio was significantly decreased, whereas that of the WMHV-to-eTIV ratio was significantly increased in the prefrail group compared with the robust group. Slowness, one of the components of physical frailty, was significantly associated with reduced TBV-to-eTIV and HV-to-eTIV ratios, and slowness and weakness were significantly associated with an increased WMHV-to-eTIV ratio. Our results suggest that the prefrail state is significantly associated with global brain atrophy and white matter hypointensities. Furthermore, slowness was significantly associated with hippocampal atrophy.

## Introduction

Physical frailty was reported to be associated with an increased risk of cognitive decline and dementia^1,2^. Physical frailty was also associated with an increased rate of decline in global cognition and cognitive components, such as episodic memory, perceptual speed, visuospatial abilities^3^, and executive function^4^. However, the etiology or mechanisms of developing dementia in people with frailty has not been clarified.

Emerging evidence suggests that physical frailty is associated with a decrease in total brain and gray matter volumes and an increase in white matter hyperintensities on fluid-attenuated inversion recovery (FLAIR)-magnetic resonance (MR) images^5–7^. Additionally, weakness and slowness, which are components of physical frailty, were associated with reduced gray matter and hippocampal volumes^8^. Therefore, physical frailty might be a prodromal stage of central nervous system vascular injury. However, individuals with physical frailty often have cognitive disorders, such as mild cognitive impairment (MCI) or dementia, and previous reports might include individuals with MCI^5–8^. Furthermore, brain atrophy might be affected by cognitive disorders. Therefore, to clarify how physical frailty affects the brain structure, the relationships between physical frailty and brain imaging findings in cognitively unimpaired older adults need to be established. In this study, we investigated that white matter lesions on the 3D T1-weighted images, whereas 2D FLAIR-MR images were often used to determine white matter lesions^5,6,9–11^. FLAIR-MR images are more sensitive for the white matter lesions than T1-weighted images, however 3D T1-weighted images may be able to detect smaller lesions than 2D FLAIR-MR images. This study aimed to examine the relationships between physical frailty and brain structure, as measured by global brain and hippocampal volumes and white matter hypointensities on 3D T1-weighted MR images among cognitively unimpaired older adults.

## Results

### Characteristics of participants

**Figure 1:**
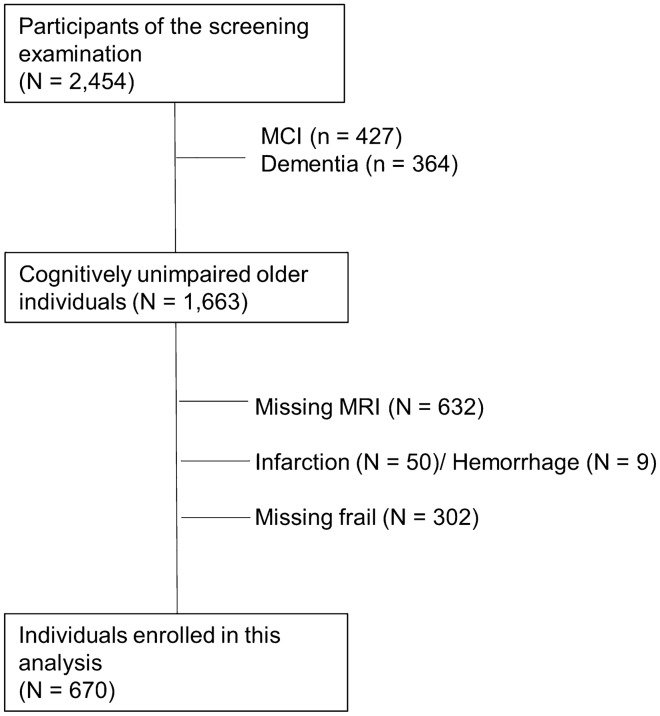
Flow chart of participant enrollment. Abbreviations: MCI, mild cognitive impairment; MRI, magnetic resonance imaging.

Compared with the individuals excluded from our analysis (n = 1784), enrolled individuals (n = 670) had significantly lower mean values for age, median values for Geriatric Depression Scale (GDS), and the proportions for hypertension, diabetes mellitus, and depressive symptoms. Conversely, the enrolled group had significantly higher mean values for serum high-density lipoprotein (HDL) cholesterol levels and for the proportions for educational level and drinking habits (Fig. 1 and Supplementary Table 1).

Of the 670 individuals, 10 were in the frail group (1.4%), whereas 241 were in the prefrail group (35.9%) (Table 1). The prevalence rates of each determinant component of the frailty phenotype, including weight loss, slowness, weakness, exhaustion, and low physical activity, were 9.7%, 8.9%, 8.8%, 2.1%, and 14.5%, respectively (Table 1). The clinical characteristics of the study population are summarized according to the physical frailty status in Table 1. The mean values for age, the median values for GDS, and the proportions of having diabetes mellitus, depressive symptoms, and smoking habits were significantly higher, whereas the mean values for serum HDL cholesterol level and the proportions for education level were significantly lower in the prefrail and frail group than in the robust group (Table 1). There were few individuals with frail group, then we mainly compared clinical characteristics and brain volumes between the individuals with prefrail and robust group.

**Table 1:** Participant characteristics by physical frailty status.

Variables	Total	Robust	Prefrail	Frail
N (%)	670 (100)	419 (62.5)	241 (36.0)	10 (1.5)
Age (y), mean (SD)	70.1 (6.4)	69.4 (5.7)	71.0 (7.3)*	78.4 (5.7)*
Women (%)	57.3	58.9	54.4	60.0
Hypertension (%)	72.7	70.2	75.9	100*
Diabetes mellitus (%)	16.2	13.6	19.9*	33.3*
Serum LDL-chol, mg/dL, mean (SD)	115.3 (30.2)	116.2 (30.2)	113.6 (30.4)	118.9 (26.7)
Serum HDL-chol, mg/dL, mean (SD)	61.2 (15.3)	62.2 (15.4)	59.3 (15.1)*	66.5 (15.6)*
Education ≤ 9 y (%)	35.5	30.5	43.2*	70.0*
Smoking habit (%)	10.6	8.4	14.5*	10.0*
Drinking habit (%)	43.5	46.1	39.0	40.0
ApoE E4, present (%)	22.7	22.2	22.4	30.0
MMSE, median (IQR)	29.0 (27.0–30.0)	29.0 (27.0–30.0)	29.0 (27.0–30.0)	28.5 (25.0–29.2)
Depressive symptoms (%)	15.5	11.5	22.4*	20.0*
GDS, median (IQR)	2.0 (1.0–4.0)	2.0 (1.0–4.0)	3.0 (1.0–5.0)*	4.0 (3.7–5.5)*
Frailty component				
Weight loss, present (%)	9.7	0	26.6	40.0
Slowness, present (%)	8.9	0	24.5	90.0
Weakness, present (%)	8.8	0	24.1	90.0
Exhaustion, present (%)	2.1	0	5.8	30.0
Low physical activity (%)	14.5	0	39.8	70.0

*SD* standard deviation, *LDL-chol* low-density lipoprotein cholesterol, *HDL-chol* high-density lipoprotein cholesterol, *MMSE* Mini-mental state examination, *IQR* interquartile range, *GDS* geriatric depression scale.

*p < 0.05, prefrail and frail group versus the robust group.

Regarding the components of the frailty phenotype, similar findings were obtained for individuals with slowness (Table 2), except for the proportion for hypertension and serum low-density lipoprotein (LDL) cholesterol level. Individuals with weakness were significantly older, had higher GDS scores, and had lower education levels, drinking habits, and Mini-Mental State Examination (MMSE) score (Table 2). Individuals with weight loss had significantly higher GDS scores. Individuals having exhaustion had a significantly higher proportion of the depressive symptoms and GDS scores. Individuals with low physical activity were significantly younger and had higher GDS scores than those in the robust group (Table 2). When we restricted the analysis to the participants having only one component of each physical frailty, individuals with slowness were significantly higher in age, proportion of diabetes mellitus, serum LDL cholesterol levels, and GDS scores. Individuals with weakness were significantly higher in age and had significantly lower education levels, drinking habits, and MMSE score than those in the robust group (Supplementary Table 2).

**Table 2:** Participant characteristics in individuals with each component of the physical frailty status among 241 individuals in the prefrail group.

Variables	Weight loss	Slowness	Weakness	Exhaustion	Low physical activity
n	64	59	58	14	96
Age (y), mean (SD)	70.0 (65.0–75.0)	78.0 (71.0–82.0)*	73.0 (69.0–81.0)*	68.5 (64.7–73.7)	66.0 (63.0–70.0)*
Women (%)	48.4	48.5	62.1	50.0	57.3
Hypertension (%)	77.8	88.1*	80.7	78.9	70.8
Diabetes mellitus (%)	21.0	30.5*	19.3	21.4	14.9
Serum LDL-chol, mg/dL, mean (SD)	112.5 (96.0–134.2)	102.0 (85.0–123.0)*	108.0 (90.0–133.0)	112.5 (86.5–149.7)	122.0 (101.0–139.2)
Serum HDL-chol, mg/dL, mean (SD)	56.0 (49.7–68.2)	55.0 (49.0–67.0)*	56.0 (50.0–70.0)	55.0 (51.2–60.5)	59.5 (50.0–68.0)
Education ≤ 9 y (%)	38.1	49.1*	56.1*	50.0	36.5
Smoking habit (%)	14.1	20.3*	6.9	21.4	21.4
Drinking habit (%)	45.3	37.3	22.4*	64.3	40.6
ApoE E4, present, (%)	22.2	18.6	20.0	28.6	22.3
MMSE, median (IQR)	29.0 (27.0–30.0)	28.0 (27.0–29.0)	28.0 (26.0–29.0)*	27.5 (26.0–29.0)	29.0 (27.0–30.0)
Depressive symptoms (%)	17.2	37.3*	20.7	42.9*	19.8
GDS, median (IQR)	3.0 (1.25–5.0)*	4.0 (2.0–6.0)*	3.5 (1.0–5.0)*	4.5 (2.0–9.0)*	3.0 (1.0–5.0)*
Frailty component					
Weight loss, present (%)	–	10.2	6.9	0	6.3
Slowness, present (%)	9.4	–	27.6*	21.4	4.2
Weakness, present (%)	6.3	27.1*	–	7.1	9.4
Exhaustion, present (%)	0	5.1	1.7	–	1.0
Low physical activity (%)	9.4	6.8	15.5	7.1	–

*SD* standard deviation, *LDL-chol* low-density lipoprotein cholesterol, *HDL-chol*, high-density lipoprotein cholesterol, *MMSE* mini-mental state examination, *IQR* interquartile range, *GDS* geriatric depression scale.

*p < 0.05 versus the robust group.

### Association of brain volume with physical frailty and its components

The age- and sex-adjusted and multivariable-adjusted mean values for the total brain volume (TBV)-to-estimated total intracranial volume (eTIV), hippocampal volume (HV)-to-eTIV, and white matter hypointensities volume (WMHV)-to-eTIV ratios are presented according to frailty status in Table 3. The prefrail group had significantly lower mean values for the TBV-to-eTIV ratio and significantly higher mean values for the WMHV-to-eTIV ratio compared with the robust group after adjustment for age, sex, hypertension, diabetes mellitus, serum LDL and HDL cholesterol levels, education level, GDS score, ApoE E4 carrier status, and MMSE score. In assessing whether the five components of physical frailty were associated with each magnetic resonance imaging (MRI) parameter, the multivariable-adjusted mean values for the TBV-to-eTIV and HV-to-eTIV ratios were significantly lower, whereas those for the WMHV-to-eTIV ratios were significantly higher in individuals with slowness compared with those in the robust group (Table 3). Additionally, the multivariable-adjusted mean values for the WMHV-to-eTIV ratio were significantly higher in individuals with weakness compared with those in the robust group (Table 3). Among ApoE E4 non-carriers, we found significant associations between prefrailty status and TBV-to-eTIV and WMHV-to-eTIV ratios, slowness and TBV-to-eTIV and WMHV-to-eTIV ratios, and a positive but insignificant association between slowness and HV-to-eTIV ratio (Supplementary Table 4). Additionally, significant associations were observed between weakness and WMHV-to-eTIV ratio and exhaustion and WMHV-to-eTIV ratio (Supplementary Table 4). When we conducted the analysis between the individuals with prefrail and frail group and robust group, these associations remained unchanged (Supplementary Table 3).

**Table 3:** Association of brain volume with prefrailty and its components.

	TBV-to-eTIV (%)		HV-to-eTIV (%)		WMHV-to-eTIV (%)	
	Age- and sex-adjusted	Multivariable-adjusted	Age- and sex-adjusted	Multivariable-adjusted	Age- and sex-adjusted	Multivariable-adjusted
**Physical frailty status**						
Robust	59.1 (58.8–59.4)	59.0 (58.6–59.5)	0.48 (0.47–0.49)	0.48 (0.47–0.49)	0.29 (0.26–0.31)	0.29 (0.25–0.33)
Prefrail	58.5 (58.1–58.9)*	58.5 (58.0–59.0)*	0.47 (0.46–0.48)	0.47 (0.47–0.48)	0.35 (0.31–0.38)*	0.35 (0.31–0.40)*
**Components of the physical frailty**						
*Weight loss*						
No	58.9 (58.7–59.2)	58.9 (58.5–59.3)	0.48 (0.47–0.48)	0.48 (0.47–0.49)	0.30 (0.28–0.33)	0.31 (0.27–0.35)
Yes	58.3 (57.6–59.0)	58.4 (57.6–59.2)	0.47 (0.46–0.48)	0.47 (0.46–0.49)	0.34 (0.27–0.41)	0.36 (0.28–0.43)
*Slowness*						
No	59.0 (58.8–59.2)	59.0 (58.6–59.4)	0.48 (0.47–0.48)	0.48 (0.47–0.49)	0.29 (0.27–0.31)	0.29 (0.26–0.33)
Yes	57.4 (56.6–58.2)*	57.4 (56.6–58.3)*	0.47 (0.46–0.48)*	0.46 (0.45–0.48)*	0.49 (0.42–0.57)*	0.49 (0.41–0.58)*
*Weakness*						
No	58.9 (58.7–59.1)	58.8 (58.4–59.2)	0.48 (0.47–0.48)	0.48 (0.47–0.49)	0.30 (0.28–0.32)	0.31 (0.27–0.35)
Yes	58.6 (57.8–59.4)	58.6 (57.7–59.4)	0.48 (0.46–0.49)	0.46 (0.45–0.49)	0.38 (0.31–0.46)*	0.40 (0.31–0.48)*
*Exhaustion*						
No	58.9 (58.6–59.1)	58.8 (58.4–59.2)	0.48 (0.47–0.48)	0.48 (0.47–0.49)	0.31 (0.28–0.33)	0.31 (0.28–0.35)
Yes	59.5 (57.9–61.0)	58.6 (58.0–61.2)	0.49 (0.46–0.52)	0.49 (0.46–0.52)	0.43 (0.28–0.58)	0.42 (0.27–0.57)
*Low physical activity*						
No	58.9 (58.7–59.2)	58.8 (58.4–59.2)	0.48 (0.47–0.47)	0.48 (0.47–0.49)	0.31 (0.28–0.33)	0.31 (0.28–0.35)
Yes	58.7 (58.1–59.3)	58.6 (57.9–59.3)	0.47 (0.46–0.48)	0.47 (0.46–0.49)	0.32 (0.26–0.38)	0.32 (0.26–0.39)

Values are shown as a mean value (95% confidence interval).

In the multivariable-adjusted model, the values were adjusted for age, sex, educational level, hypertension, diabetes mellitus, LDL and HDL cholesterol levels, ApoE E4 carrier status, MMSE score, and GDS score.

*Benjamini–Hochberg false discovery rate-adjusted *p* value, *q* < 0.05.

Furthermore, we investigated whether the five components of physical frailty were associated with each MRI-extracted brain trait among individuals having only one component of each physical frailty (Table 4). Individuals with slowness had significantly lower multivariable-adjusted mean values for the TBV-to-eTIV and HV-to-eTIV ratios and higher values for the WMHV-to-eTIV ratio than those in the robust group (Table 4).

**Table 4:** Association of Brain Volume with Physical Frailty and its Components among individuals with any one component of the physical frailty status.

	TBV-to-eTIV (%)		HV-to-eTIV (%)		WMHV-to-eTIV (%)	
	Age- and sex- adjusted	Multivariable-adjusted	Age- and sex- adjusted	Multivariable-adjusted	Age- and sex- adjusted	Multivariable-adjusted
**Any one component of physical frailty**						
Robust	59.2 (59.0–59.5)	59.0 (58.6–59.4)	0.48 (0.48–0.49)	0.48 (0.47–0.49)	0.28 (0.26–0.30)	0.29 (0.25–0.32)
Any one component of the physical frailty	58.7 (58.3–59.1)*	58.6 (58.1–59.1)	0.48 (0.47–0.48)	0.48 (0.47–0.49)	0.30 (0.27–0.33)	0.31 (0.27–0.35)
**Components of physical frailty**						
*Weight loss*						
No	59.1 (58.8–59.3)	58.9 (58.5–59.2)	0.48 (0.48–0.48)	0.48 (0.47–0.49)	0.29 (0.27–0.31)	0.30 (0.26–0.33)
Yes	58.8 (58.1–59.6)	58.9 (58.1–59.7)	0.48 (0.46–0.49)	0.48 (0.47–0.50)	0.27 (0.20–0.33)	0.28 (0.21–0.36)
*Slowness*						
No	59.1 (58.9–59.3)	59.0 (58.6–59.3)	0.48 (0.48–0.49)	0.48 (0.47–0.49)	0.28 (0.26–0.30)	0.28 (0.25–0.31)
Yes	57.9 (56.9–58.8)*	57.9 (56.9–58.8)*	0.45 (0.44–0.47)*	0.46 (0.44–0.48)*	0.46 (0.38–0.54)*	0.46 (0.37–0.54)*
*Weakness*						
No	59.1 (58.9–59.3)	58.9 (58.6–59.3)	0.48 (0.47–0.48)	0.48 (0.47–0.49)	0.29 (0.27–0.30)	0.30 (0.26–0.33)
Yes	58.7 (57.7–59.6)	58.5 (57.4–59.6)	0.48 (0.46–0.50)	0.48 (0.46–0.51)	0.28 (0.20–0.37)	0.29 (0.20–0.39)
*Exhaustion*						
No	59.0 (58.8–59.2)	58.9 (58.5–59.2)	0.48 (0.47–0.48)	0.48 (0.47–0.49)	0.29 (0.27–0.30)	0.29 (0.26–0.33)
Yes	60.3 (58.5–62.0)	60.3 (58.5–62.0)	0.50 (0.46–0.53)	0.50 (0.47–0.53)	0.32 (0.17–0.47)	0.31 (0.16–0.46)
*Low physical activity*						
No	59.1 (58.9–59.3)	58.9 (58.6–59.3)	0.48 (0.48–0.48)	0.48 (0.47–0.49)	0.29 (0.27–0.31)	0.30 (0.27–0.33)
Yes	58.7 (58.1–59.3)	58.5 (57.9–59.2)	0.48 (0.46–0.49)	0.48 (0.46–0.49)	0.27 (0.22–0.32)	0.27 (0.22–0.33)

Values were shown as a mean value (95% confidence interval).

In the multivariable-adjusted model, the values were adjusted for age, sex, educational levels, hypertension, diabetes mellitus, LDL and HDL cholesterol levels, ApoE E4 carrier status, MMSE score, and GDS score.

*Benjamini–Hochberg false discovery rate-adjusted *p* value, *q* < 0.05.

## Discussion

Our study demonstrated for the first time that the prefrail state and the components of physical frailty, such as slowness and weakness, were significantly associated with brain structural changes in cognitively unimpaired older adults. The prefrail state was significantly associated with global brain atrophy and white matter hypointensities on 3D T1-weighted imaging. Slowness was associated with global brain atrophy, reduced hippocampal volumes, and white matter hypointensities, whereas weakness was associated with white matter hypointensities. When we analyzed the participants among ApoE E4 non-carriers, we found a significant association between prefrail state and global brain atrophy and white matter hypointensities, and a positive but insignificant association was observed between slowness and reduced hippocampal volumes. This study found that the prefrail state, which is early-stage physical frailty, is associated with brain structural changes in cognitively unimpaired older adults.

Our analysis revealed that slowness was significantly associated with hippocampal atrophy. Individuals with slowness but no other components of frailty had significantly lower hippocampal volumes than those in the robust group. The longitudinal or cross-sectional association of hippocampal atrophy with gait speed decline has also been reported^12,13^ and agrees with our present findings. A randomized controlled trial showed that aerobic exercise training increases the size of the hippocampus, leading to improvements in spatial memory^14^. Furthermore, gait speed and cognitive impairment may be partly due to a shared underlying neuropathology, and hippocampal atrophy appears to be associated with both^15^. In rodents, it was reported that exercise enhances learning, which is accompanied by increased cell proliferation and survival in the hippocampus by increased production and secretion of brain-derived neurotrophic factor (BDNF)^16^. Also in human, exercise training increased hippocampal volume which was associated with greater serum levels of BDNF^14^. Gait slowness related to inactivity may cause hippocampal atrophy by decreased level of BDNF. Further longitudinal studies are needed to clarify the mechanisms in the development of hippocampal atrophy and slowness prior to the onset of cognitive decline. Although frailty is thought to be a reversible state^17^, its pathophysiology has not been fully understood. The hippocampal neurons could be easily affected by changes in synaptic and mitochondrial functions^18^. Increasing physical activity may have long-term positive effects on hippocampal volume^19^. The potential reversibility of physical frailty and hippocampal atrophy could be clarified in future investigations.

White matter hypointensities on 3D T1-weighted imaging are a marker of cerebral small vessel disease^20^ and may be associated with an increased risk of vascular dementia. This study showed for the first time that the prefrailty state and especially the component of slowness were associated with white matter hypointensities. These associations remained unchanged in the analysis among the participants with ApoE E4 non-carriers. Conflicting results have been reported regarding the association between physical frailty and white matter hypointensities on 3D T1-weighted imaging and white matter hyperintensities on FLAIR scans^3,6,9–11^. Some studies have shown a significant association between physical frailty and white matter hyperintensities^5,6,11^, which matches our present findings. In contrast, some studies found no association between physical frailty and white matter hyperintensities^9,10^. These conflicting results may be related to a difference in the assessment criteria for physical frailty. Three previous studies used the physical frailty phenotype^5,9,11^, one used the Frailty Index^6^, and another one used the Edmonton Frail Scale^10^. Further large-scale longitudinal studies are needed to clarify the association between physical frailty and white matter hypointensities on 3D T1-weighted imaging.

The limitations of this study should be addressed. First, as the findings of this study were derived from cross-sectional data, establishing a causal association between physical frailty and structural brain abnormalities was difficult. Second, we could not perform the analyses for the frail group because of the small sample size of participants classified as frail.

The study findings showed that the prefrail state was significantly associated with global brain atrophy and cerebral small vessel disease in cognitively unimpaired general older adults. Additionally, physical frailty, as indicated by slowness, was significantly associated with hippocampal atrophy and cerebral small vessel disease. Further prospective longitudinal studies and basic research are required to verify the findings of this study.

## Methods

### Study population

The Nakajima study is an ongoing population-based longitudinal cohort study that investigates cognitive decline in older Japanese individuals. The study was conducted in Nakajima, in Nanao City of Ishikawa Prefecture, Japan. The study design has been described previously^21^. From 2016 to 2018, a total of 2454 residents aged 60 years or older (92.9% of the total population in this age group) participated in the screening examination for dementia. To clarify whether frailty is a risk factor for cognitive impairment among cognitively unimpaired older adults, we excluded patients with dementia (n = 364) and MCI (n = 427). MCI and dementia were confirmed using the clinical criteria defined by Petersen et al.^22^ and the Third Diagnostic and Statistical Manual of Mental Disorders, Revised Edition (DSM-III-R)^23^. We also excluded individuals who had hemorrhagic and/or ischemic stroke lesions on MRI regardless of the presence or absence of neurological symptoms (n = 59), no brain MRI (n = 632), or no physical frailty assessments (n = 302) (Fig. 1). Hemorrhagic and/or ischemic stroke lesions were pointed from two trained neuro-radiologists who were blinded to the clinical information. Individuals with terrible hypertension (blood pressure levels ≥ 180/110 mmHg) and individuals with leg pain during gait were excluded from physical frailty assessments.

Finally, 670 individuals were enrolled for analysis (286 male, 384 female; age, mean [standard deviation, SD] is 70.1 [6.4] years).

### Standard protocol approvals and participant consent

This study was conducted in accordance with the guidelines of the Declaration of Helsinki and all procedures were approved by the Medical Ethics Review Board of Kanazawa University (Approval Number 2185). We obtained written informed consent from all participants.

### MRI analysis

The structural MRI studies were performed using a 1.5 T system (ECHELON RX; Hitachi, Japan). A 3D volumetric acquisition of a T1-weighted turbo field echo images was conducted according to the brain MRI protocol for the Alzheimer’s Disease Neuroimaging Initiative (ADNI) study^24^ (Echo time/Repetition time, 4.0/9.2 ms; flip angle, 8°; Field of View, 240 mm; acquisition matrix, 192 × 192; number of slices, 170; voxel size, 0.9375 × 0.9375 mm; slice thickness, 1.2 mm). All T1-structural images were analyzed using FreeSurfer version 5.3 (FreeSurfer version 5.3; http://surfer.nmr.mgh.harvard.edu)^25^ in Tohoku University and preprocessed according to the standard manner. The volumes of the area of interest were created by FreeSurfer^26^, and the HV and WMHV were calculated as the sum of the volumes of the right and left hippocampi and white matter hypointensities, respectively. TBV was calculated by summing the white and gray matter volumes and eTIV was used to normalize each volumetric value. In this study, we evaluated three parameters, namely, the TBV-to-eTIV ratio (%), HV-to-eTIV ratio (%), and WMHV-to-eTIV ratio (%), to determine the potential indices of global brain atrophy, hippocampal atrophy, and severity of cerebral small vessel disease, respectively.

### Assessment of physical frailty

This study assessed physical frailty and its phenotype according to the Fried criteria^14^, namely, weight loss, slowness, weakness, exhaustion, and low physical activity. We collected data from 2016 to 2018 and assessed physical frailty using the revised Japanese version of the Cardiovascular Health Study criteria (revised J-CHS criteria)^27^. except for exhaustion.

Weight loss was assessed by responses to the self-reported question “Have you (unintentionally) lost 2 kg or more in the past 6 months?” (“Yes” = 1 point). Slowness was defined as a comfortable gait speed < 1.0 m/s (1 point). Weakness was defined as a maximum grip strength < 28 kg in men and < 18 kg in women (1 point). Exhaustion was assessed by responses to the self-reported questions “Did you feel that everything you did was an effort?” and “Did you feel exhausted without any reason?” (“Yes” to either = 1 point) using the operational definition of exhaustion in the Sasaguri Genkimon Study^28^. Low physical activity was assessed by responses to the self-reported questions “Do you do low levels of physical exercise?” and “Do you do moderate levels of physical exercise or sports?” (“No” to both = 1 point). Based on these definitions, we defined the frail group as having 3 points or more, the prefrail group as having 1 or 2 points, and the robust group as 0 point. Since only 10 people were in the frail group, we analyzed the frail group together with the prefrail group.

### Other risk factor measurements

Each participant completed a self-administered questionnaire that contains questions on sociodemographic data (age, sex, and educational level), medical history (diabetes mellitus and hypertension), drug information, smoking, and drinking habits. The completed questionnaires were reviewed by researchers trained to identify inconsistent or unanswered items. Depressive symptoms were evaluated using the GDS–Short Form^29^, and a GDS score of ≥ 6 was used to denote depressive symptoms. Blood pressure was measured three times using a sphygmomanometer, with an interval of at least 5 min. The average of the three measurements was used for further analysis. Hypertension was determined by blood pressure levels ≥ 140/90 mmHg or the current use of antihypertensive agents. Body mass index (BMI, kg/m^2^) was measured as an indicator of obesity. Serum high-density lipoprotein (HDL) and low-density lipoprotein (LDL) cholesterol levels were enzymatically measured^30^. The ApoE phenotype was determined using isoelectric electrophoresis as described by Kamboh et al.^31^.

### Statistical analyses

Clinical characteristics of each frailty status were compared using the t-test for the mean values of continuous variables, the Mann–Whitney U test for median values for the MMSE and GDS, and the chi-squared test for continuous and categorical variables. A two-tailed *p* < 0.05 was considered statistically significant. Analysis of covariance was used to estimate and compare the age- and sex-adjusted or multivariable-adjusted values and their 95% confidence intervals for the TBV-to-eTIV, HV-to-eTIV, and WMHV-to-eTIV ratios. In the multivariable-adjusted analysis, age, sex, educational level, hypertension, diabetes mellitus status, LDL and HDL cholesterol levels, ApoE E4 carrier status, MMSE score, and GDS score were analyzed as covariates. The problem of multiple comparisons was solved by controlling the Benjamini–Hochberg false discovery rate (FDR)^32^. The FDR-adjusted *p* values (i.e., *q* values) below 0.05 were considered statistically significant. The SPSS software package (version 26; SPSS Inc., Chicago, IL, USA) was used to perform all statistical analyses.

## Supplementary Information

Supplementary Information.

## Data Availability

The datasets used in the current study are not publicly available, because they contain confidential clinical data on the study participants. However, the data are available on reasonable request and with the permission of the corresponding author, Moeko Noguchi-Shinohara (Department of Neurology and Neurobiology of Aging, Kanazawa University Graduate School of Medical Sciences, Kanazawa, Japan).
