# Supplementary material for: Association of the prefrailty with global brain atrophy and white matter lesions among cognitively unimpaired older adults: the Nakajima study
Source: Sci Rep. 2022 Aug 1;12:12129. doi: 10.1038/s41598-022-16190-7 (PMC9343640; doi:10.1038/s41598-022-16190-7)
Supplement: Supplementary file 1 — Supplementary Information. [file 41598_2022_16190_MOESM1_ESM.docx]

**Supplementary Table 1. Characteristics of the enrolled and excluded individuals in this study**

| **Variables** | **Enrolled** | **Excluded** |
| --- | --- | --- |
| n (%) | 670 (27.3) | 1784 (72.6) |
| Age (y), mean (SD) | 70.1 (6.4) | 77.1 (9.3)* |
| Women, % | 57.3 | 57.8 |
| Hypertension, % | 72.7 | 87.7* |
| Diabetes mellitus, % | 16.2 | 42.9* |
| Serum LDL-chol, mg/dL, mean (SD) | 115.3 (30.2) | 113.8 (35.4) |
| Serum HDL-chol, mg/dL, mean (SD) | 61.2 (15.3) | 58.8 (15.1)* |
| Education ≤ 9 y, % | 35.5 | 62.6* |
| Smoking habit, % | 10.6 | 11.0 |
| Drinking habit, % | 43.5 | 33.1* |
| ApoE E4, present, % | 22.7 | 22.1 |
| MMSE, median (IQR) | 29.0 (27.0–30.0) | 26.0 (22.0–28.0)* |
| Depressive symptoms, % | 15.5 | 23.0 |
| GDS, median (IQR) | 2.0 (1.0–4.0) | 3.0 (1.0–5.0)* |

**p* < 0.05.

Abbreviations: SD, standard deviation; LDL-chol, low-density lipoprotein cholesterol; HDL-chol, high-density lipoprotein cholesterol; MMSE, Mini-Mental State Examination; IQR, interquartile range; GDS, Geriatric Depression Scale.

**Supplementary Table 2. Participant Characteristics by Physical Frailty Status in the 150 individuals with any one of the physical frailty components**

| **Variables** | **Having 1 component of physical frailty** | **Weight loss** | **Slowness** | **Weakness** | **Exhaustion** | **Low physical activity** |
| --- | --- | --- | --- | --- | --- | --- |
| n (%) | 191 | 48 | 30 | 28 | 9 | 76 |
| Age (y), mean (SD) | 70.2 (6.8) | 70.8 (6.4) | 75.4 (7.8)* | 72.6 (6.6)* | 68.3 (3.5) | 67.1 (5.4)* |
| Women, % | 55.0 | 50.0 | 53.3 | 64.3 | 55.6 | 55.3 |
| Hypertension, % | 74.1 | 74.5 | 83.3 | 74.1 | 77.8 | 69.7 |
| Diabetes mellitus, % | 19.9 | 17.4 | 36.7* | 18.5 | 22.2 | 14.9 |
| Serum LDL-chol, mg/dL, mean (SD) | 113.6 (31.0) | 114.7 (29.7) | 102.8 (28.5)* | 110.7 (32.1) | 118.4 (32.8) | 117.7 (31.8) |
| Serum HDL-chol, mg/dL, mean (SD) | 60.1 (15.6) | 60.5 (12.9) | 57.7 (11.6) | 63.5 (18.8) | 56.1 (20.6) | 60.0 (16.8) |
| Education ≤ 9 y, % | 42.9 | 41.7 | 50.0 | 57.1* | 55.6 | 34.2 |
| Smoking habit, % | 13.1 | 8.3 | 16.7 | 7.1 | 11.1 | 17.1* |
| Drinking habit, % | 39.8 | 47.9 | 36.7 | 17.9* | 44.4 | 43.4 |
| ApoE E4, present, % | 25.4 | 27.7 | 23.3 | 28.0 | 22.2 | 24.3 |
| MMSE, median (IQR) | 29.0 (27.0–30.0) | 29.0 (27.0–30.0) | 28.5 (27.0–29.2) | 27.5 (25.0–29.0)* | 28.0 (25.5–29.0) | 29.0 (27.0–30.0) |
| Depressive symptoms, % | 19.9 | 20.8 | 26.7 | 7.1 | 33.3 | 19.7 |
| GDS, median (IQR) | 3.0 (1.0–5.0) | 3.0 (2.0–5.0)* | 3.0 (1.0–6.0)* | 2.0 (1.0–5.0) | 4.0 (2.5–7.5)* | 3.0 (1.0–5.0)* |

**p* < 0.05 versus the robust group.

Abbreviations: SD, standard deviation; LDL-chol, low-density lipoprotein cholesterol; HDL-chol, high-density lipoprotein cholesterol; MMSE, Mini-Mental State Examination; IQR, interquartile range; GDS, Geriatric Depression Scale.

**Supplementary Table 3.** **Association of Brain Volume with Physical Frailty and its Components**

|  | **TBV-to-eTIV (%)** | |  | **HV-to-eTIV (%)** | |  | **WMHV-to-eTIV (%)** | |
| --- | --- | --- | --- | --- | --- | --- | --- | --- |
|  | Age- and sex- adjusted | Multivariable-adjusted |  | Age- and sex- adjusted | Multivariable-adjusted |  | Age- and sex- adjusted | Multivariable-adjusted |
| **Physical frailty status** | |  |  |  |  |  |  |  |
| Robust | 59.1 (58.8–59.4) | 59.0 (58.6–59.4) |  | 0.48 (0.47–0.48) | 0.48 (0.47–0.49) |  | 0.29 (0.26–0.32) | 0.30 (0.26–0.36) |
| Prefrail and frail | 58.4 (58.1–58.8)* | 58.4 (57.9–58.9)* |  | 0.47 (0.46–0.48) | 0.47 (0.46–0.48) |  | 0.35 (0.32–0.39)* | 0.36 (0.311–0.41)* |
| **Components of physical frailty** | |  |  |  |  |  |  |  |
| ***Weight loss*** |  |  |  |  |  |  |  |  |
| No | 58.9 (58.6–59.1) | 58.8 (58.4–59.2) |  | 0.48 (0.47–0.48) | 0.48 (0.47–0.49) |  | 0.31 (0.29–0.33) | 0.32 (0.28–0.36) |
| Yes | 58.4 (57.7–59.1) | 58.5 (57.7–59.3) |  | 0.47 (0.46–0.48) | 0.48 (0.46–0.49) |  | 0.33 (0.26–0.40) | 0.35 (0.27–0.43) |
| ***Slowness*** |  |  |  |  |  |  |  |  |
| No | 59.0 (58.8–59.2) | 59.0 (58.6–59.3) |  | 0.48 (0.47–0.48) | 0.48 (0.47–0.49) |  | 0.29 (0.27–0.31) | 0.30 (0.26–0.34) |
| Yes | 57.4 (56.6–58.1)* | 57.4 (56.6–58.2)* |  | 0.46 (0.44–0.47)* | 0.46 (0.45–0.48)* |  | 0.50 (0.43–0.57)* | 0.51 (0.43–0.59)* |
| ***Weakness*** |  |  |  |  |  |  |  |  |
| No | 58.9 (58.6–59.1) | 58.8 (58.4–59.2) |  | 0.48 (0.47–0.48) | 0.48 (0.47–0.49) |  | 0.30 (0.28–0.33) | 0.31 (0.27–0.35) |
| Yes | 58.4 (57.7–59.1) | 58.3 (57.5–59.2) |  | 0.47 (0.46–0.48) | 0.47 (0.46–0.49) |  | 0.40 (0.33–0.47)* | 0.42 (0.34–0.50)* |
| ***Exhaustion*** |  |  |  |  |  |  |  |  |
| No | 58.8 (58.6–59.1) | 58.8 (58.4–59.2) |  | 0.48 (0.47–0.48) | 0.48 (0.47–0.48) |  | 0.31 (0.29–0.33) | 0.32 (0.28–0.36) |
| Yes | 59.4 (57.0–59.8) | 58.6 (57.0–60.1) |  | 0.48 (0.45–0.50) | 0.49 (0.46–0.51) |  | 0.44 (0.30–0.57) | 0.45 (0.30–0.59) |
| ***Low physical activity*** |  |  |  |  |  |  |  |  |
| No | 58.9 (58.6–59.1) | 58.8 (58.4–59.2) |  | 0.48 (0.47–0.48) | 0.48 (0.47–0.49) |  | 0.31 (0.29–0.33) | 0.32 (0.28–0.36) |
| Yes | 58.7 (58.1–59.3) | 58.6 (57.9–59.3) |  | 0.47 (0.46–0.48) | 0.47 (0.46–0.49) |  | 0.33 (0.37–0.38) | 0.33 (0.27–0.40) |
| Values were shown as a mean value (95% confidence interval).  In the multivariable-adjusted model, the values were adjusted for age, sex, educational levels, hypertension, diabetes mellitus, LDL and HDL cholesterol levels, ApoE E4 carrier status, MMSE score, and GDS score. | | | | | | | | |

*Benjamini–Hochberg false discovery rate-adjusted *p* value, *q* < 0.05.

**Supplementary Table 4. Association of Brain Volume with Prefrailty and its Components among ApoE E4 non-carriers**

|  | **TBV-to-eTIV (%)** | |  | **HV-to-eTIV (%)** | |  | **WMHV-to-eTIV (%)** | |
| --- | --- | --- | --- | --- | --- | --- | --- | --- |
|  | Age- and sex-adjusted | Multivariable-adjusted |  | Age- and sex-adjusted | Multivariable-adjusted |  | Age- and sex-adjusted | Multivariable-adjusted |
| **Physical frailty status** | |  |  |  |  |  |  |  |
| Robust | 59.0 (58.7–59.4) | 59.0 (58.6–59.5) |  | 0.48 (0.47–0.48) | 0.48 (0.47–0.49) |  | 0.29 (0.25–0.32) | 0.30 (0.25–0.35) |
| Prefrail | 58.4 (57.9–58.8)* | 58.4 (57.8–58.9)* |  | 0.47 (0.46–0.48) | 0.47 (0.47–0.48) |  | 0.37 (0.33–0.41)* | 0.38 (0.33–0.43)* |
| **Components of the physical frailty** | |  |  |  |  |  |  |  |
| ***Weight loss*** |  |  |  |  |  |  |  |  |
| No | 58.8 (58.6–59.1) | 58.8 (58.4–59.3) |  | 0.48 (0.47–0.48) | 0.48 (0.47–0.49) |  | 0.31 (0.28–0.34) | 0.33 (0.28–0.37) |
| Yes | 58.2 (57.3–59.0) | 58.3 (57.3–59.2) |  | 0.47 (0.46–0.49) | 0.47 (0.46–0.49) |  | 0.36 (0.28–0.45) | 0.39 (0.29–0.48) |
| ***Slowness*** |  |  |  |  |  |  |  |  |
| No | 58.9 (58.7–59.2) | 59.0 (58.5–59.4) |  | 0.48 (0.47–0.48) | 0.48 (0.47–0.49) |  | 0.30 (0.27–0.32) | 0.31 (0.27–0.35) |
| Yes | 57.3 (56.4–58.2)* | 57.3 (56.3–58.3)* |  | 0.46 (0.45–0.48) | 0.47 (0.45–0.48) |  | 0.51 (0.42–0.60)* | 0.52 (0.43–0.62)* |
| ***Weakness*** |  |  |  |  |  |  |  |  |
| No | 58.8 (58.5–59.1) | 58.8 (58.4–59.3) |  | 0.48 (0.47–0.48) | 0.48 (0.47–0.49) |  | 0.31 (0.28–0.34) | 0.32 (0.28–0.36) |
| Yes | 58.5 (57.6–59.4) | 58.4 (57.4–59.4) |  | 0.47 (0.46–0.49) | 0.48 (0.46–0.49) |  | 0.42 (0.33–0.51)* | 0.43 (0.33–0.53)* |
| ***Exhaustion*** |  |  |  |  |  |  |  |  |
| No | 58.8 (58.5–59.0) | 58.8 (58.3–59.2) |  | 0.48 (0.47–0.48) | 0.48 (0.47–0.49) |  | 0.31 (0.29–0.34) | 0.33 (0.29–0.37) |
| Yes | 59.3 (57.4–61.2) | 59.2 (57.3–61.2) |  | 0.48 (0.44–0.51) | 0.48 (0.45–0.51) |  | 0.52 (0.33–0.71)* | 0.52 (0.33–0.71)* |
| ***Low physical activity*** |  |  |  |  |  |  |  |  |
| No | 58.8 (58.5–59.1) | 58.8 (58.4–59.2) |  | 0.48 (0.47–0.48) | 0.48 (0.47–0.49) |  | 0.31 (0.28–0.34) | 0.33 (0.29–0.37) |
| Yes | 58.7 (57.9–59.4) | 58.6 (57.8–59.4) |  | 0.47 (0.46–0.48) | 0.48 (0.46–0.49) |  | 0.34 (0.27–0.41) | 0.34 (0.27–0.42) |
| Values are shown as a mean value (95% confidence interval).  In the multivariable-adjusted model, the values were adjusted for age, sex, educational level, hypertension, diabetes mellitus, LDL and HDL cholesterol levels, ApoE E4 carrier status, MMSE score, and GDS score. | | | | | | | | |

*Benjamini–Hochberg false discovery rate-adjusted *p* value, *q* < 0.05.
